# The sweet spot: fasting glucose, cardiovascular disease, and mortality in older adults with diabetes: a nationwide population-based study

**DOI:** 10.1186/s12933-020-01021-8

**Published:** 2020-04-01

**Authors:** Ji Hyun Lee, Kyungdo Han, Ji Hye Huh

**Affiliations:** 1 Cardiovascular Center, Department of Internal Medicine, Seoul National University Bundang Hospital, Seongnam, South Korea; 2 Department of Medical Statistics, College of Medicine, The Catholic University of Korea, Seoul, South Korea; 3 Division of Endocrinology and Metabolism, Department of Internal Medicine, Hallym University Sacred Heart Hospital, Anyang, 220-701 South Korea

**Keywords:** Cardiovascular events, Diabetes, Fasting glucose, Mortality, Older adults

## Abstract

**Background:**

Growing evidences shows that fasting glucose target should be different according to their health condition in older adults with diabetes. However, there are limited data regarding the relationship between fasting glucose level and health outcomes in Korean older people with diabetes. We aimed to examine the association of fasting glucose with mortality and cardiovascular events in Korean older adults with type 2 diabetes.

**Methods:**

From the Korean National Health Insurance System, 227,938 subjects (aged ≥ 65 years) with type 2 diabetes but no history of cardiovascular events (myocardial infarction or stroke) who underwent ≥ 2 health examinations from 2009 to 2010 and who were followed up until 2017 were identified. The primary exposure variable was the mean fasting glucose level. We estimated the relationship between the baseline fasting glucose level and incidences of all-cause death and cardiovascular events. Comorbidity load was assessed using the Charlson comorbidity index.

**Results:**

Fasting glucose levels and all-cause mortality risk showed a J-shaped relationship regardless of sex and number of comorbidities. Fasting glucose levels associated with the lowest mortality and cardiovascular events were 110–124 and 95–124 mg/dL, respectively. Stratified analysis by comorbidity load using the Charlson comorbidity index revealed higher optimal fasting glucose levels for the lowest cardiovascular events in subjects with Charlson comorbidity index ≥ 3 than in those with Charlson comorbidity index ≤ 2 (119 vs. 112 mg/dL, P = 0.04).

**Conclusions:**

J-shaped relationship existed between fasting glucose and all-cause mortality and cardiovascular events in Korean older adults with diabetes. We identified that fasting glucose levels associated with the lowest mortality and cardiovascular events were 110–124 and 95–124 mg/dL respectively. Increased risk of cardiovascular events with low fasting glucose level (< 95 mg/dL) was noted, especially in patients with high comorbidity. These findings suggested that less stringent targets of fasting glucose may be beneficial especially in older adults with multiple comorbidities.

## Background

Diabetes in individuals aged ≥ 65 years has globally become a growing public health burden. Type 2 diabetes is an age-related disease with an increasing prevalence of 33% in the US population aged ≥ 65 years [1]. In Korea, nearly 30% of older people meet the criteria for diabetes [2]. The fact that the prevalence of diabetes and diabetes-related complications, such as myocardial infarction (MI) and ischemic stroke, are increasing in older age groups [3], emphasizes the importance of glycemic control in these populations. However, the management of older adults with diabetes is clearly more complicated because they commonly have multiple comorbidities that can impact the clinical management. Therefore, healthcare for older adults with type 2 diabetes must consider this complexity when setting or prioritizing treatment goals [4].

Good glycemic control is associated with a lower risk of mortality and cardiovascular (CV) events in people with diabetes. Fasting glucose level is a fundamental element in managing diabetes to achieve good glycemic control. Some observational studies have shown J-shaped distributions for mortality and glycemic control, and both high and low fasting glucose levels are associated with a higher risk of mortality [5, 6]. The results of several observational and randomized control studies assessing glucose lowering strategies and health outcomes and current guidelines emphasize individualized and less stringent glycemic targets for older people with diabetes [7]. Furthermore, current guidelines suggest different glycemic target levels according to the underlying chronic conditions and comorbidities in these individuals. However, the benefits and risks of fasting glycemic thresholds especially in older East Asian adults with type 2 diabetes are unclear.

Using large-scale nationwide cohort data from the Korean population, we aimed to identify the optimal fasting glucose range associated with the lowest risk of mortality and CV events in older adults with type 2 diabetes. We also examined whether the optimal fasting glucose ranges for health outcomes differ according to comorbidities in Korean people with diabetes aged ≥ 65 years.

## Methods

### Study design and subjects

We used data from the National Health Insurance Service-National Health Screening (NHIS-HEALS) cohort in Korea. The insurance system was established by the Korean government and covers approximately 97% of residents. Subjects aged ≥ 40 years are entitled to a general health screening program every 2 years. The screening includes standardized self-reporting questionnaires on medical history and lifestyle behaviors, anthropometric and blood pressure measurements, and routine laboratory tests using blood and urine. Thus, NHIS-HEALS would be nationally representative cohort database consisting of nearly the whole South Korean population. The cohort profile of the NHIS-HEALS has been described previously [8]. Among individuals who underwent health examination provided by the NHIS at least once between January 1, 2009 and December 31, 2010, we excluded those with no prior diagnosis of diabetes (n = 15,819,273). The occurrence of diabetes mellitus was defined according to the following criteria: (i) at least two claims under International Classification of Diseases 10th Revision (ICD-10) codes or (ii) fasting glucose level ≥ 126 mg/dL. We further excluded subjects with a diagnosis of MI or stroke (n = 71,691) prior to enrollment and those aged ≤ 64 years (n = 1168,473). We also excluded those with any missing data on fasting glucose (n = 1953). To minimize bias caused by the single fasting glucose measurement, we used the mean fasting glucose value obtained from the 2009–2010 and 2011–2012 measurements. Thus, subjects who did not participate in the second health screening in 2011–2012 (n = 250,664) were excluded. The remaining 227,938 eligible individuals (110,123 men and 117,815 women) whose fasting glucose were checked twice at baseline, were included in the analyses and followed up until the date of death or until December 31, 2017. The flow chart for the selection process is shown in Additional file 1. This study was approved by the institutional review board of Yonsei University Wonju College of Medicine (CR318331). The requirement for written informed consent was waived by the review board because anonymous and de-identified information was used for analysis.

### Measurements and definitions

Information on current smoking and alcohol consumption was obtained using a questionnaire. Heavy alcohol consumption was defined as ≥ 5 or ≥ 4 alcoholic drinks per week for males and females, respectively. Regular exercise was defined as physical activity that was performed at least five times per week. Blood samples for the measurement of serum fasting glucose and cholesterol levels were drawn after an overnight fast. Hospitals wherein these health examinations were performed were certified by the NHIS and subjected to regular quality control. Baseline comorbidities (hypertension, dyslipidemia, congestive heart failure, chronic obstructive pulmonary disease, depression, chronic kidney disease, and dementia) were identified based on the combination of past medical history, ICD-10 Clinical Modification (ICD-10-CM), and prescription codes. To assess the overall comorbidity load, we used the primary care equivalent of the Charlson comorbidity index (CCI) [9]. The CCI provides a way of quantifying this impact in terms of survival and is also used as a prognostic comorbidity index. CCI was developed empirically 26 years ago as a prognostic index of comorbid conditions for patients admitted for general medical service with a variety of medical conditions. Such conditions, alone or in combination, might alter the risk of short-term mortality in patients enrolled in longitudinal studies.

### Outcomes and exposures

The endpoints of this study were newly diagnosed CV events (MI or ischemic stroke) and all-cause mortality. MI was defined as ICD-10 codes I21 or I22 during hospitalization or when these codes were recorded at least two times. Ischemic stroke was defined as ICD-10 codes I63 or I64 during hospitalization with claims for brain magnetic resonance imaging or brain computerized tomography [10]. Mortality data were extracted from the death statistics from the Korean National Statistical Office. Subjects without MI or stroke during follow-up periods were considered to have completed the study at the date of their death or at the end of follow-up, whichever came first. The study population was followed up from the date of the second fasting glucose measurement in 2011–2012 to the date of death or CV events, or until December 31, 2017, whichever came first.

### Statistical analysis

Data are expressed as means with standard deviations, n (%), or hazard ratios (HRs) with 95% confidence intervals (CIs). We compared means in two groups using the Student’s t test and compared categorical variables using the Chi-square test. Subjects were classified into ten groups according to the fasting glucose levels at increasing intervals of 15 mg/dL. The risk of overall mortality and CV events (MI or stroke) was calculated using Cox proportional hazards model with fasting glucose level as the dependent variable. In addition, to reduce the impact of competing risk bias on the result, we performed competing risk model analysis to assess the risk of CVD, with death considered as a competing event, using subdistribution hazard model by Fine-Gray [11]. The following covariates: age, sex, income, place of living (urban vs. rural), smoking, duration of diabetes, alcohol, exercise, body mass index, systolic blood pressure, CCI, and cholesterol were included in the multivariable model. The incidence rate of outcomes was calculated by dividing the number of incidences by the total follow-up duration (person-years). A restricted cubic spline transformation of fasting glucose was used to evaluate nonlinear associations. In the subgroup analysis, the presence of interaction was assessed in multivariate models by testing the significance of two-way interaction terms.

Statistical analyses were performed using SAS version 9.4 (SAS Institute Inc., Cary, NC, USA), and a P-value < 0.05 indicated statistical significance.

## Results

### Baseline characteristics

Overall, 227,938 subjects were included in the analysis. The mean age of the study subjects was 72.5 years (Additional file 2). Of the study subjects, males were 48% (n = 110,123), while in about 67% of patients, the diabetes duration was ≥ 5 years. Hypertension and dyslipidemia prevalence were 74% and 46%, respectively. About 61% of the study population had CCI ≥ 3, while almost all the subjects (94%) were receiving pharmacological treatment for diabetes.

### Risk of all-cause mortality according to fasting glucose level

**Fig. 1:**
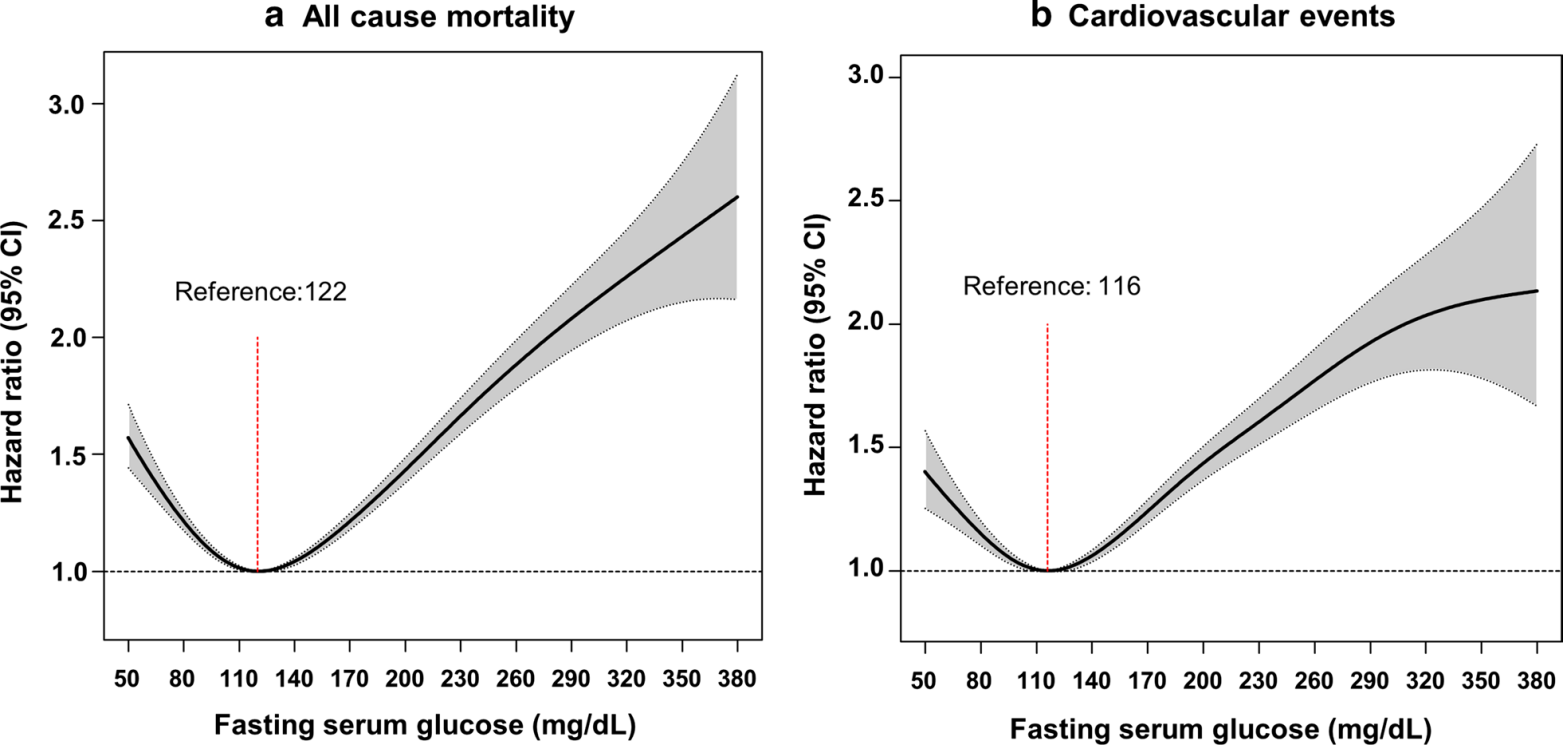
Hazard ratios for all-cause mortality (**a**) and cardiovascular events (**b**) according to fasting glucose levels at baseline. *Hazard ratios were calculated by Cox models after adjusting for age at baseline, sex, family income, residential area, smoking status, diabetes duration (≥ 5 years/< 5 year), alcohol intake, regular exercise, body mass index, systolic blood pressure, Charlson comorbidity index and total cholesterol

Overall, in this cohort, 27,212 deaths occurred in a median of 5 years (mean 5.7 ± 1.1 years) of the follow-up period. The fasting glucose levels and the risk of all-cause mortality showed a J-shaped relationship, with both the too low and high fasting glucose levels showing a higher risk of mortality (Fig. 1a). The optimal glucose level associated with the lowest risk of all-cause mortality was 122 mg/dL in cubic spline analysis. In categorical analysis, the glucose range of 110–124 mg/dL was associated with the lowest risk of all-cause mortality (Table 1).

**Table 1:** Hazard ratios and 95% confidence intervals of all-cause mortality, cardiovascular events (myocardial infarction or stroke) by 10 categories of fasting glucose level at baseline

Event	Fasting glucose (mg/dL)	Number of subjects	Number of event	Follow-up duration (person-years)	Incident rate (per 1000 person-years)	Crude hazard ratio (95% confidence interval)	Adjusted hazard ratio^a^ (95% confidence interval)
All-cause mortality	≤ 79	1913	349	10,637.92	32.81	1.85 (1.66–2.06)	1.54 (1.38–1.72)
	80–94	13,151	1857	74,421.57	24.95	1.40 (1.33–1.48)	1.25 (1.19–1.32)
	95–109	36,921	4238	211,544.25	20.03	1.12 (1.08–1.17)	1.08 (1.04–1.13)
	110–124	51,028	5260	293,999.64	17.89	1 (reference)	1 (reference)
	125–139	50,469	5373	290,389.59	18.50	1.03 (0.99–1.07)	1.06 (1.02–1.11)
	140–154	31,763	3671	182,174.08	20.15	1.13 (1.08–1.18)	1.16 (1.11–1.21)
	155–169	17,381	2227	99,423.1	22.40	1.25 (1.19–1.32)	1.25 (1.19–1.31)
	170–184	10,067	1416	57,331.8	24.70	1.38 (1.31–1.47)	1.35 (1.27–1.43)
	185–199	6008	992	33,796.48	29.35	1.65 (1.54–1.77)	1.60 (1.49–1.71)
	≥ 200	9237	1829	51,389.79	35.59	2.01 (1.90–2.12)	1.89 (1.79–1.99)

^a^Hazard ratios were calculated by Cox models after adjusting for age at baseline, sex, family income, residential area, smoking status, diabetes duration (≥ 5 years/< 5 year), alcohol intake, regular exercise, body mass index, systolic blood pressure, Charlson comorbidity index and total cholesterol

Multivariable adjusted HRs (95% CI) of mortality associated with fasting glucose levels < 80, 80–95, 95–110, 125–140, 140–155, 155–170, 170–185, 185–200, and > 200 mg/dL were 1.54 (1.38–1.72), 1.25 (1.19–1.32), 1.08 (1.04–1.13), 1.06 (1.02–1.11), 1.16 (1.11–1.21), 1.25 (1.19–1.31), 1.35 (1.27–1.43), 1.60 (1.49–1.71), and 1.89 (1.79–2.0) respectively, compared with the 110–124-mg/dL level.

### Risk of cardiovascular events according to the fasting glucose level

During the mean 5.6 ± 1.2 years of follow-up, 17,406 newly diagnosed CV events (6398 MI and 11,863 ischemic stroke) occurred. The relationship between fasting glucose and CV events also showed a J-shaped distribution in study subjects. The fasting glucose level associated with the lowest CV event risk was 116 mg/dL in cubic spline analysis (Fig. 1b). Among the different fasting glucose level categories, those ranging between 95 and 124 mg/dL were associated with the lowest risk of CV events. Multivariable adjusted HRs (95% CI) of CV events associated with fasting glucose levels of < 80, 80–95, 95–110, 125–140, 140–155, 155–170, 170–185, 185–200, and > 200 mg/dL were 1.27 (1.10–1.48), 1.14 (1.06–1.22), 0.99 (0.94–1.05), 1.05 (1.01–1.10), 1.12 (1.06–1.18), 1.25 (1.17–1.32), 1.36 (1.27–1.46), 1.47 (1.35–1.60), and 1.76 (1.64–1.88) respectively, compared with the 110–124 mg/dL level (Table 2). Similar pattern was observed in the competing risk model analysis (Additional file 3).

**Table 2:** Hazard ratios and 95% confidence intervals of cardiovascular events (myocardial infarction or stroke) by 10 categories of fasting glucose level at baseline

Event	Fasting glucose (mg/dL)	Number of subjects	Number of event	Follow-up duration (person-years)	Incident rate (per 1000 person-years)	Crude hazard ratio (95% confidence interval)	Adjusted hazard ratio^a^ (95% confidence interval)
Cardiovascular events	≤ 79	1913	178	10,196.6	17.46	1.41 (1.21–1.64)	1.27 (1.10–1.48)
	80–94	13,151	1063	71,565.21	14.85	1.20 (1.12–1.29)	1.14 (1.06–1.22)
	95–109	36,921	2545	204,466.84	12.45	1.01 (0.96–1.06)	0.99 (0.94–1.05)
	110–124	51,028	3503	284,218.61	12.33	1 (reference)	1 (reference)
	125–139	50,469	3540	280,507.05	12.62	1.03 (0.98–1.07)	1.05 (1.01–1.10)
	140–154	31,763	2392	175,497.47	13.63	1.11 (1.05, 1.17)	1.12 (1.06–1.18)
	155–169	17,381	1492	95,226.41	15.67	1.27 (1.20–1.35)	1.25 (1.17–1.32)
	170–184	10,067	949	54,660.29	17.36	1.41 (1.31–1.51)	1.36 (1.27–1.46)
	185–199	6008	613	32,133.93	19.08	1.55 (1.42–1.69)	1.47 (1.35–1.60)
	≥ 200	9237	1131	48,310.17	23.41	1.89 (1.77–2.03)	1.76 (1.64–1.88)

^a^Hazard ratios were calculated by Cox models after adjusting for age at baseline, sex, family income, residential area, smoking status, diabetes duration (≥ 5 years/< 5 year), alcohol intake, regular exercise, body mass index, systolic blood pressure, Charlson comorbidity index and total cholesterol

### Interaction of comorbidity on the relationship between fasting glucose and adverse event

**Fig. 2:**
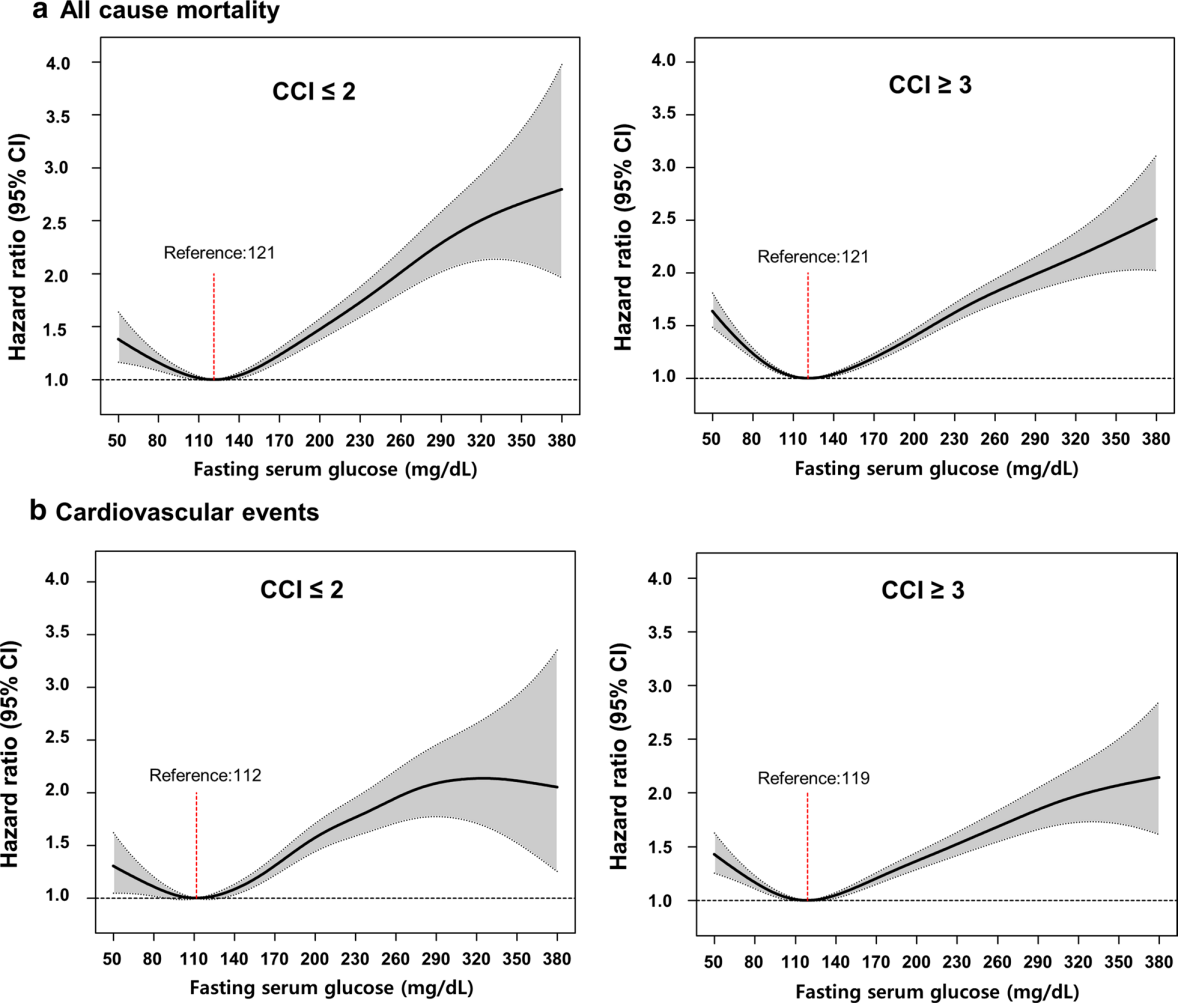
Hazard ratios for all-cause mortality (**a**) cardiovascular events (**b**) according to fasting glucose levels at baseline stratified by Charlson comorbidity index (≤ 2, ≥ 3). *CCI* Charlson comorbidity index; *Hazard ratios were calculated by Cox models after adjusting for age at baseline, sex, family income, residential area, smoking status, diabetes duration (≥ 5 years/< 5 year), alcohol intake, regular exercise, body mass index, systolic blood pressure, Charlson comorbidity index and total cholesterol

When the analysis was separately performed in subjects with low (CCI ≤ 2) and high (CCI ≥ 3) comorbidity load, J-shaped relationship between fasting glucose and all-cause mortality was observed in both groups (Fig. 2a). The optimal fasting glucose levels associated with the lowest risk of all-cause mortality were 121 mg/dL in subjects with both CCI ≤ 2 and ≥ 3 in cubic spline analysis. Patients with fasting glucose levels of 110–124 mg/dL showed the lowest risk for all-cause mortality among the different fasting glucose level categories in both comorbidity groups (Table 3). There was no significant difference in the trend of mortality risk between the two groups (*P* for interaction = 0.12).

**Table 3:** Hazard ratios and 95% confidence intervals of all-cause mortality by 10 categories of fasting glucose level at baseline stratified by Charlson co-morbidity index (CCI)

All-cause mortality								
	Fasting glucose (mg/dL)	Number of subjects	Number of event	Follow-up duration (person-years)	Incident rate (per 1000 person-years)	Crude hazard ratio (95% confidence interval)	Adjusted hazard ratio^a^ (95% confidence interval)	P for interaction
CCI (≤ 2)	≤ 79	579	72	3316.07	21.71	1.6 (1.26–2.03)	1.33 (1.05–1.68)	0.12
	80–94	4344	432	25,071.35	17.23	1.27 (1.14–1.41)	1.17 (1.06–1.31)	
	95–109	13,120	1148	76,002.87	15.10	1.11 (1.03–1.20)	1.09 (1.01–1.18)	
	110–124	19,268	1527	112,249.59	13.60	1 (reference)	1 (reference)	
	125–139	21,556	1808	125,243.71	14.44	1.06 (0.99–1.14)	1.04 (0.97–1.12)	
	140–154	13,930	1286	80,764.71	15.92	1.17 (1.09–1.26)	1.14 (1.06–1.23)	
	155–169	7284	752	42,089.97	17.87	1.32 (1.21–1.44)	1.25 (1.14–1.36)	
	170–184	4046	460	23,372.6	19.68	1.45 (1.31–1.61)	1.41 (1.27–1.56)	
	185–199	2349	336	13,361.23	25.15	1.86 (1.65–2.09)	1.71 (1.52–1.92)	
	≥ 200	3484	557	19,744.85	28.21	2.09 (1.90–2.30)	1.96 (1.77–2.16)	
CCI (≥ 3)	≤ 79	1334	277	7321.85	37.83	1.86 (1.65–2.11)	1.59 (1.41–1.80)	
	80–94	8807	1425	49,350.22	28.88	1.41 (1.33–1.50)	1.28 (1.20–1.36)	
	95–109	23,801	3090	135,541.38	22.80	1.11 (1.06–1.17)	1.08 (1.03–1.14)	
	110–124	31,760	3733	181,750.05	20.54	1 (reference)	1 (reference)	
	125–139	28,913	3565	165,145.88	21.59	1.05 (1.00–1.10)	1.07 (1.02–1.12)	
	140–154	17,833	2385	101,409.37	23.52	1.15 (1.09–1.21)	1.15 (1.09–1.21)	
	155–169	10,097	1475	57,333.12	25.73	1.25 (1.18–1.33)	1.23 (1.16–1.31)	
	170–184	6021	956	33,959.21	28.15	1.37 (1.28–1.48)	1.32 (1.23–1.42)	
	185–199	3659	656	20,435.25	32.10	1.57 (1.45–1.71)	1.53 (1.40–1.66)	
	≥ 200	5753	1272	31,644.94	40.20	1.98 (1.85–2.11)	1.84 (1.73–1.97)	

^a^Hazard ratios were calculated by Cox models after adjusting for age at baseline, sex, family income, residential area, smoking status, diabetes duration (≥ 5 years/< 5 year), alcohol intake, regular exercise, body mass index, systolic blood pressure, and total cholesterol

When assessing the risk of CV events, similar trends were observed. The optimal fasting glucose levels associated with the lowest risk of CV events were 112 mg/dL and 119 mg/dL in subjects with CCI ≤ 2 and ≥ 3, respectively, in cubic spline analysis (Fig. 2b). Among subjects with CCI ≤ 2, those with fasting glucose level below the optimal value (< 112 mg/dL) showed a gentle slope curve with wide CI compared with those with CCI ≥ 3 who showed a steeper slope of HR curve with less wide CI. When assessing the risk of CV events according to the different categories of fasting glucose, the group with a fasting glucose level of 95–124 mg/dL showed the lowest risk of CV events in both comorbidity groups (Table 4).

**Table 4:** Hazard ratios and 95% confidence intervals of cardiovascular events (myocardial infarction or stroke) by 10 categories of fasting glucose level at baseline stratified by Charlson co-morbidity index (CCI)

Cardiovascular events								
	Fasting glucose (mg/dL)	Number of subjects	Number of event	Follow-up duration (person-years)	Incident rate (per 1000 person-years)	Crude hazard ratio (95% confidence interval)	Adjusted hazard ratio^a^ (95% confidence interval)	P for interaction
CCI (≤ 2)	≤ 79	579	36	3231.06	11.14	1.17 (0.84–1.63)	1.09 (0.78–1.51)	0.04
	80–94	4344	250	24,425.72	10.24	1.07 (0.93–1.23)	1.04 (0.91–1.19)	
	95–109	13,120	715	74,074.88	9.65	1.01 (0.92–1.11)	1.00 (0.91–1.10)	
	110–124	19,268	1050	109,502.23	9.59	1 (reference)	1 (reference)	
	125–139	21,556	1230	121,947.97	10.09	1.05 (0.97–1.14)	1.05 (0.97–1.14)	
	140–154	13,930	895	78,409.27	11.41	1.19 (1.09–1.30)	1.16 (1.06–1.27)	
	155–169	7284	529	40,701.97	13.00	1.36 (1.22–1.51)	1.29 (1.16–1.44)	
	170–184	4046	338	22,453.28	15.05	1.57 (1.39–1.78)	1.51 (1.34–1.71)	
	185–199	2349	204	12,856.68	15.87	1.66 (1.43–1.93)	1.54 (1.33–1.79)	
	≥ 200	3484	366	18,811.37	19.46	2.04 (1.81–2.29)	1.90 (1.69–2.14)	
CCI (≥ 3)	≤ 79	1334	142	6965.54	20.39	1.44 (1.22–1.71)	1.32 (1.11–1.56)	
	80–94	8807	813	47,139.49	17.25	1.22 (1.13–1.33)	1.16 (1.07–1.26)	
	95–109	23,801	1830	130,391.96	14.03	1.00 (0.94–1.06)	0.99 (0.93–1.05)	
	110–124	31,760	2453	174,716.38	14.04	1 (reference)	1 (reference)	
	125–139	28,913	2310	158,559.08	14.57	1.04 (0.98–1.10)	1.05 (0.99–1.11)	
	140–154	17,833	1497	97,088.2	15.42	1.10 (1.03–1.17)	1.09 (1.02–1.16)	
	155–169	10,097	963	54,524.43	17.66	1.26 (1.17–1.35)	1.21 (1.13–1.31)	
	170–184	6021	611	32,207.01	18.97	1.35 (1.24–1.48)	1.29 (1.18–1.41)	
	185–199	3659	409	19,277.25	21.22	1.51 (1.36–1.67)	1.42 (1.28–1.58)	
	≥ 200	5753	765	29,498.8	25.93	1.83 (1.69–1.99)	1.68 (1.55–1.82)	

^a^Hazard ratios were calculated by Cox models after adjusting for age at baseline, sex, family income, residential area, smoking status, diabetes duration (≥ 5 years/< 5 year), alcohol intake, regular exercise, body mass index, systolic blood pressure, and total cholesterol

However, in subjects with CCI ≤ 2, the risk of CV events among those with fasting glucose level ≤ 94 mg/dL was not significantly higher than that among those with fasting glucose level of 110–124 mg/dL. A significant comorbidity interaction was noted (*P *= 0.04).

## Discussion

In this study, we examined the optimal range of fasting glucose levels associated with the lowest risk of mortality and CV events in Korean older adults with type 2 diabetes using a nationwide database. We found J-curved associations between fasting glucose and clinical outcomes, including all-cause mortality and CV events, in these individuals. The optimal range of fasting glucose levels associated with the lowest risk of all-cause mortality was 110–124 mg/dL. The optimal range associated with the lowest risk of CV events was 95–124 mg/dL. This trend was observed consistently in both sexes (Additional file 4). Although the overall associations and fasting glucose range for lower mortality were similar for those with lower (CCI ≤ 2) and higher (CCI ≥ 3) comorbidity load, the optimal fasting glucose level associated with the lowest CV events was higher in those with a higher (CCI ≥ 3) comorbidity load than in those with a lower (CCI ≤ 2) load (119 vs. 112 mg/dL). Furthermore, the risk of CV events increased much more, as the fasting glucose decreased below the optimal fasting glucose level in those with CCI ≥ 3 compared to those with CCI < 2. This finding suggests that less stringent targets of fasting glucose may be favorable for CV outcome in older adults with multiple comorbidities. To our knowledge, this is the first nationwide study to investigate the optimal fasting glucose level associated with the lowest risk of adverse events in older East Asian adults with diabetes.

Consistent with our study, several previous studies have demonstrated the J-shaped relationship between fasting glucose level and adverse outcomes, including all-cause mortality and CV events, not only in people with diabetes but also in the healthy population [12–15]. Recent meta-analyses and a nationwide observational study revealed an increased risk of CV event in adults with pre-diabetes even in fasting glucose range (100–125 mg/dL) [16, 17]. On the contrary, our study showed that the fasting glucose range of 95–124 mg/dL seemed to be optimal for the prevention of CV events in patients with diabetes. Although the suggested optimal fasting glucose levels or ranges associated with the lowest risk of adverse outcomes vary (from 80 to 109 mg/dL) because of the different baseline characteristics in each study population, it is obvious that the risk of adverse events increases as the fasting glucose level deviates outside the optimal range [12–14]. The optimal fasting glucose levels for better health outcomes seem to be increased as patients become older or as diabetes progresses [13]. Since our study enrolled only older people with diabetes, the optimal fasting glucose range associated with the lowest risk of adverse events (about 95–124 mg/dL) could be higher than that reported in previous studies. These findings further support that the optimal cut-off point of the fasting glucose level may be different in the general population and in those with co-morbidities.

Interestingly, our study showed that the fasting glucose level associated with the lowest risk of all-cause mortality was higher than that associated with the lowest risk of CV events (122 vs. 116 mg/dL). Furthermore, the present study demonstrated a slightly increased risk of mortality in patients with even normal fasting glucose level (80–109 mg/dL) who did not have the hypoglycemic status. However, these observations are congruent with the finding of a previous randomized control study conducted in the Western population. In the Action to Control Cardiovascular Risk in Type 2 Diabetes trial, compared with standard therapy group, the intensive glycemic control group showed a higher incidence of all-cause mortality. Meanwhile, the incidence of composite CV outcomes of non-fatal MI, non-fatal stroke, and cardiac death was numerically fewer in the intensive glycemic control group [18]. Although the exact mechanism to explain this phenomenon could not be established in our study, we speculate that more stringent glycemic control in older adults was associated with a higher risk of severe hypoglycemia; consequently, this may result in higher number of deaths directly due to hypoglycemia [18, 19]. Regarding the conflicting impact of glucose levels of 95–110 mg/dL on mortality and CV events observed in our study, a more extensive study is warranted.

In our study, there was an increasing risk of mortality or CV events with fasting glucose values < 95 mg/dL or ≥ 125 mg/dL. These findings provide further evidence that not only too loose glucose control but also too stringent glucose control could increase the risk of adverse health outcomes in older patients with diabetes. There is substantial evidence indicating that too strict glucose control induces severe hypoglycemia and increases the risk of mortality and CV events [20, 21]. Hypoglycemia induces sympatho-adrenal activation and counter-regulatory hormonal secretion [22]. This condition can consequently trigger cardiac arrhythmia, vasoconstriction, thrombogenicity, vascular inflammation, and vasoconstriction, leading to cardiovascular event or mortality [23]. Although the optimal range for fasting blood glucose associated with lower risk, as suggested in our study (95–124 mg/dL), might be narrower than that suggested by the global guideline, it overlaps with the general target range of fasting glucose (90–130 mg/dL) recommended by a global diabetes guideline for older adults with diabetes [24].

The global guidelines recommend less stringent glycemic control in older adults with diabetes (aged ≥ 65 years) especially those with many comorbidities and impaired functionalities [7, 25]. Likewise, our study demonstrated that as the fasting glucose level decreases below the optimal range, much steeper increases in the risk of adverse events were noted in subjects with higher comorbidity (CCI ≥ 3) compared with those with lower comorbidity (CCI ≤ 2). This finding indicates that a more careful glycemic control is needed in fragile patients with both diabetes and multiple comorbidities to avoid hypoglycemia. In the American Diabetes Association guideline, the control level of fasting glucose within 90–150 mg/dL was recommended in older patients with both diabetes and multiple existing chronic illnesses, including MI, heart failure, and stroke [7]. Our suggested optimal fasting glucose range (110–124 mg/dL) associated with the lowest risk of mortality in patients with high comorbidity (CCI ≥ 3) falls under this target range of fasting glucose. Considering the narrow window with an abrupt increase in the risk of adverse event at the outer range of fasting glucose of 110–124 mg/dL, the proposed wide target range of fasting glucose (90–150 mg/dL) in the guideline might be suboptimal for our study population. However, we should not directly compare these target values because our subjects were relatively healthier persons without previous history of stroke or MI.

A major strength of the present study is that we used national data that included the entire Korean population. All detailed reimbursement claim data were searched. However, there were several limitations to this study. First, we used only fasting glucose level as a surrogate marker for the intensity of glycemic control because HbA1c measurement was not included the national health screening program in our country during the study period. However, the use of HbA1c as a marker for glycemic control also has some limitations, especially in patients with medical conditions that impact the red blood cell turnover. These medical conditions are commonly seen in older adults [26]. Second, fasting glucose measurement may show wide within-person variation. To minimize the bias caused by a single measurement of fasting glucose level, we used the mean fasting glucose level measured in both 2009–2010 and 2011–2012. Furthermore, glycemic variability, a strong predictor of CV events [27–30], could not be assessed because of the limited instance of fasting glucose measurements, in the present study. Third, the controlled fasting glucose levels during the future follow-up period could not be predicted. Fourth, in the categorical analysis, fasting glucose ranges were artificially set at every 15-mg/dL increase. Thus, the true optimal glucose ranges could be slightly different from those in this study. Fifth, we could not collect data on hypoglycemic episodes in the study population. However, when we assessed the risk after further adjustment for anti-hyperglycemic agents (including insulin secretagogue or insulin) that may cause hypoglycemic episodes, we observed similar pattern of results with those of models where anti-hyperglycemic agents were not adjusted for (Additional file 5). Finally, the specific cause of mortality could not be evaluated in our study.

## Conclusion

In conclusion, J-shaped relationship existed between the fasting glucose levels and adverse events, including all-cause mortality and CV events, in Korean older adults with diabetes. The optimal fasting glucose range associated with the lowest risk of all-cause mortality and CV events was 110–124 and 95–124 mg/dL, respectively. Furthermore, steep increase in the risk of adverse events in the low fasting glucose range was noted in patients with high comorbidity load. Thus, there is need for special caution in this population, in terms of glycemic control.

## Supplementary information

**Additional file 1.** Study population.

**Additional file 2.** Characteristics of participants.

**Additional file 3.** Hazard ratios and 95% confidence intervals of cardiovascular event according to the 10 categories of fasting glucose level at baseline, estimated by Fine-Gray regression.

**Additional file 4.** Hazard ratios and 95% confidence intervals of all-cause mortality (A), cardiovascular events (myocardial infarction or stroke) (B) by 10 categories of fasting glucose level stratified by sex.

**Additional file 5.** Hazard ratios and 95% confidence intervals of all-cause mortality, and CVD by 10 categories of fasting glucose level at baseline.

## Data Availability

The datasets generated and analyzed during the current study are not publicly available due to rule of Korea National health insurance system.

## Abbreviations

CCI: Charlson comorbidity index
CI: Confidence intervals
CV: Cardiovascular
FG: Fasting glucose
HRs: Hazard ratios
ICD-10: International Classification of Diseases 10th Revision
MI: Myocardial infarction
NHIS-HEALS: National Health Insurance Service-National Health Screening
