# Supplementary figures and images for: The sweet spot: fasting glucose, cardiovascular disease, and mortality in older adults with diabetes: a nationwide population-based study
Source: Cardiovasc Diabetol. 2020 Apr 1;19:44. doi: 10.1186/s12933-020-01021-8 (PMC7110776; doi:10.1186/s12933-020-01021-8)

**Additional file 1. Study population.**

**
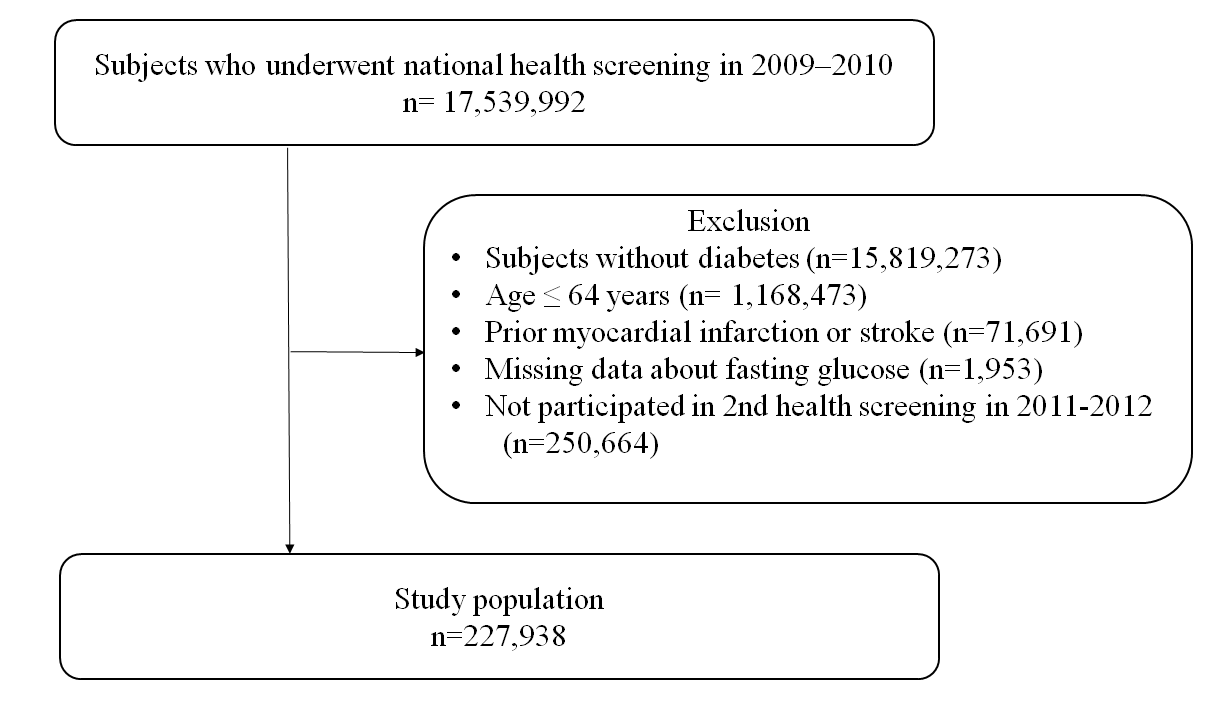
**

Supplement: Supplementary file 1 — Additional file 1. Study population. [file 12933_2020_1021_MOESM1_ESM.docx]
