# Supplementary material for: The sweet spot: fasting glucose, cardiovascular disease, and mortality in older adults with diabetes: a nationwide population-based study
Source: Cardiovasc Diabetol. 2020 Apr 1;19:44. doi: 10.1186/s12933-020-01021-8 (PMC7110776; doi:10.1186/s12933-020-01021-8)
Supplement: Supplementary file 2 — Additional file 2. Characteristics of participants. [file 12933_2020_1021_MOESM2_ESM.docx]

**Additional file 2. Characteristics of participants.**

|  | | **Total** | **Male** | **Female** |
| --- | --- | --- | --- | --- |
| **n** | | **227938** | **110123** | **117815** |
| **Age** | | 72.51 ± 4.16 | 72.32 ± 4.11 | 72.69 ± 4.21 |
| **Duration of diabetes (≥5 years)** | | 153543 (67.36) | 73423 (66.67) | 80120 (68) |
| **Body mass index(kg/m^2^)** | | 24.53 ± 3.07 | 24.18 ± 2.81 | 24.86 ± 3.27 |
| **Regular exercise** | | 91135 (39.98) | 53646 (48.71) | 37489 (31.82) |
| **Smoker** | |  |  |  |
| Never | | 162142 (71.13) | 47568 (43.2) | 114574 (97.25) |
| Ex-smoker | | 42478 (18.64) | 41319 (37.52) | 1159 (0.98) |
| Current | | 23318 (10.23) | 21236 (19.28) | 2082 (1.77) |
| **Alcohol consumption** | |  |  |  |
| Non | | 173816 (76.26) | 60984 (55.38) | 112832 (95.77) |
| Mild | | 46445 (20.38) | 41582 (37.76) | 4863 (4.13) |
| Heavy | | 7677 (3.37) | 7557 (6.86) | 120 (0.1) |
| **Hypertension** | | 169102 (74.19) | 79911 (72.57) | 89191 (75.7) |
| **Dyslipidemia** | | 104852 (46) | 41133 (37.35) | 63719 (54.08) |
| **Congestive heart failure** | | 8153 (3.58) | 3324 (3.02) | 4829 (4.1) |
| **Chronic obstructive pulmonary disease** | | 33363 (14.64) | 17228 (15.64) | 16135 (13.7) |
| **Depression** | | 19962 (8.76) | 6892 (6.26) | 13070 (11.09) |
| **Chronic kidney disease** | | 23901 (10.49) | 9775 (8.88) | 14126 (11.99) |
| **Dementia** | 27602 (12.11) | | 10451 (9.49) | 17151 (14.56) |
| **Charlson comorbidity index (CCI)** |  | |  |  |
| 0-2 | 89960 (39.5) | | 46121 (41.9) | 43839 (37.2) |
| ≥3 | 137978 (60.5) | | 64002(58.1) | 73976 (62.8) |
| **DM Medication** | 213453 (93.65) | | 101124 (91.83) | 112329 (95.34) |
| Insulin | 27339 (11.99) | | 13166 (11.96) | 14173 (12.03) |
| Sulfonylurea | 152880 (67.07) | | 73099 (66.38) | 79781 (67.72) |
| Metformin | 164847 (72.32) | | 77777 (70.63) | 87070 (73.9) |
| Meglitinides | 5442 (2.39) | | 2776 (2.52) | 2666 (2.26) |
| Thiazolidinedione | 16025 (7.03) | | 7808 (7.09) | 8217 (6.97) |
| DPP-4 inhibitor | 44324 (19.45) | | 20973 (19.05) | 23351 (19.82) |
| Acarbose | 39027 (17.12) | | 19230 (17.46) | 19797 (16.8) |
| **Systolic blood pressure (mmHg)** | 131.03 ± 15.63 | | 130.51 ± 15.36 | 131.51 ± 15.86 |
| **Fasting blood glucose (mg/dL)** | 131.57 ± 38.29 | | 133.22 ± 38.9 | 130.02 ± 37.64 |
| **Total Cholesterol (mg/dL)** | 183.07 ± 38.74 | | 176.59 ± 36.32 | 189.13 ± 39.93 |
| **Triglyceride(mg/dL)** | 129.06 (128.79 - 129.33) | | 124.77 (124.39 - 125.16) | 133.21 (132.83 - 133.58) |
| **HDL-C(mg/dL)** | 50.15 ± 13.41 | | 48.56 ± 13.32 | 51.63 ± 13.32 |
| **LDL-C(mg/dL)** | 104.09 ± 34.49 | | 100.04 ± 32.53 | 107.86 ± 35.81 |

Data are n(%) or mean ± standard deviation or median (interquartile range)

DPP-4, Dipeptidylpeptidase-4; DM, diabetes mellitus; HDL-C, high-density lipoprotein cholesterol; LDL-C, low-density lipoprotein cholesterol
