# Supplementary material for: The sweet spot: fasting glucose, cardiovascular disease, and mortality in older adults with diabetes: a nationwide population-based study
Source: Cardiovasc Diabetol. 2020 Apr 1;19:44. doi: 10.1186/s12933-020-01021-8 (PMC7110776; doi:10.1186/s12933-020-01021-8)
Supplement: Supplementary file 3 — Additional file 3. Hazard ratios and 95% confidence intervals of cardiovascular event according to the 10 categories of fasting glucose level at baseline, estimated by Fine-Gray regression. [file 12933_2020_1021_MOESM3_ESM.docx]

**Additional file 3. Hazard ratios and 95% confidence intervals of cardiovascular event according to the 10 categories of fasting glucose level at baseline, estimated by Fine-Gray regression.**

| Event | Fasting Glucose (mg/dL) | Number of event | Follow-up duration  (person-years) | Incident rate (per 1,000 person-years) | Adjusted hazard ratio*  (95% confidence interval) |
| --- | --- | --- | --- | --- | --- |
| Cardiovascular event |  |  |  |  |  |
|  | ≤79 | 349 | 10637.92 | 32.81 | 1.25 (1.07-1.45) |
|  | 80-94 | 1857 | 74421.57 | 24.95 | 1.12 (1.05-1.20) |
|  | 95-109 | 4238 | 211544.25 | 20.03 | 1.00 (0.94-1.04) |
|  | 110-124 | 5260 | 293999.64 | 17.89 | 1 (reference) |
|  | 125-139 | 5373 | 290389.59 | 18.50 | 1.05 (1.01- 1.10) |
|  | 140-154 | 3671 | 182174.08 | 20.15 | 1.10 (1.04-1.16) |
|  | 155-169 | 2227 | 99423.1 | 22.40 | 1.23 (1.16-1.31) |
|  | 170-184 | 1416 | 57331.8 | 24.70 | 1.34 (1.24-1.44) |
|  | 185-199 | 992 | 33796.48 | 29.35 | 1.42 (1.30-1.55) |
|  | ≥200 | 1829 | 51389.79 | 35.59 | 1.67 (1.56-1.79) |

*adjustment for age at baseline, sex, family income, residential area, smoking status, diabetes duration (≥5 years/< 5 year), alcohol intake, regular exercise, body mass index, systolic blood pressure, Charlson comorbidity index and total cholesterol
