# Supplementary material for: The sweet spot: fasting glucose, cardiovascular disease, and mortality in older adults with diabetes: a nationwide population-based study
Source: Cardiovasc Diabetol. 2020 Apr 1;19:44. doi: 10.1186/s12933-020-01021-8 (PMC7110776; doi:10.1186/s12933-020-01021-8)
Supplement: Supplementary file 4 — Additional file 4. Hazard ratios and 95% confidence intervals of all-cause mortality (A), cardiovascular events (myocardial infarction or stroke) (B) by 10 categories of fasting glucose level stratified by sex. [file 12933_2020_1021_MOESM4_ESM.docx]

**Additional file 4**. **Hazard ratios and 95% confidence intervals of all-cause mortality (A), cardiovascular events (myocardial infarction or stroke) (B) by 10 categories of fasting glucose level stratified by sex.**

1. All-cause mortality

| Sex | Fasting Glucose (mg/dL) | Number of subjects | Number of death | Follow-up duration  (person-years) | Incident rate (per 1,000 person-years) | Crude hazard ratio  (95% confidence interval) | Adjusted hazard ratio*  (95% confidence interval) |
| --- | --- | --- | --- | --- | --- | --- | --- |
| Male | ≤80 | 924 | 212 | 4994.3 | 42.45 | 1.70 (1.48, 1.95) | 1.40 (1.22, 1.61) |
|  | 80-95 | 5682 | 1116 | 31216.97 | 35.75 | 1.43 (1.33, 1.53) | 1.21 (1.13, 1.29) |
|  | 95-110 | 16121 | 2641 | 90162.84 | 29.29 | 1.16 (1.10, 1.22) | 1.09 (1.04, 1.15) |
|  | 110-125 | 23329 | 3333 | 131809.4 | 25.29 | 1 (reference) | 1 (reference) |
|  | 125-140 | 25340 | 3545 | 143252.74 | 24.75 | 0.98 (0.93, 1.03) | 1.05 (1.00, 1.10) |
|  | 140-155 | 16484 | 2421 | 92941.37 | 26.05 | 1.03 (0.98, 1.09) | 1.12 (1.06, 1.18) |
|  | 155-170 | 9057 | 1419 | 51050.12 | 27.80 | 1.10 (1.03, 1.17) | 1.17 (1.10, 1.24) |
|  | 170-185 | 5261 | 921 | 29404.7 | 31.32 | 1.24 (1.15, 1.34) | 1.28 (1.19, 1.38) |
|  | 185-200 | 3087 | 630 | 17023.27 | 37.01 | 1.47 (1.35, 1.60) | 1.51 (1.38, 1.64) |
|  | ≥200 | 4838 | 1143 | 26375.43 | 43.34 | 1.73 (1.62, 1.85) | 1.73 (1.62, 1.85) |
| Female | ≤80 | 989 | 137 | 5643.62 | 24.28 | 2.06 (1.73, 2.45) | 1.78 (1.50, 2.12) |
|  | 80-95 | 7469 | 741 | 43204.6 | 17.15 | 1.45 (1.33, 1.58) | 1.34 (1.23, 1.46) |
|  | 95-110 | 20800 | 1597 | 121381.41 | 13.16 | 1.11 (1.04, 1.19) | 1.08 (1.01, 1.15) |
|  | 110-125 | 27699 | 1927 | 162190.25 | 11.88 | 1 (ref.) | 1 (ref.) |
|  | 125-140 | 25129 | 1828 | 147136.85 | 12.42 | 1.05 (0.98, 1.11) | 1.09 (1.02, 1.16) |
|  | 140-155 | 15279 | 1250 | 89232.72 | 14.01 | 1.18 (1.10, 1.27) | 1.21 (1.13, 1.30) |
|  | 155-170 | 8324 | 808 | 48372.98 | 16.70 | 1.41 (1.30, 1.53) | 1.39 (1.28, 1.51) |
|  | 170-185 | 4806 | 495 | 27927.11 | 17.72 | 1.49 (1.35, 1.65) | 1.47 (1.33, 1.62) |
|  | 185-200 | 2921 | 362 | 16773.21 | 21.58 | 1.82 (1.63, 2.04) | 1.74 (1.55, 1.94) |
|  | ≥200 | 4399 | 686 | 25014.36 | 27.42 | 2.33 (2.13, 2.54) | 2.17 (1.99, 2.37) |

(B) Cardiovascular event

| Fasting Glucose (mg/dL) | Number of subjects | Number of event | Follow-up duration  (person-years) | Incident rate (per 1,000 person-years) | Crude hazard ratio  (95% confidence interval) | Adjusted hazard ratio*  (95% confidence interval) |
| --- | --- | --- | --- | --- | --- | --- |
| Male | ≤ 80 | 87 | 4789.61 | 18.1643 | 1.33 (1.08, 1.65) | 1.21 (0.97, 1.50) |
|  | 80-95 | 489 | 29964.2 | 16.3195 | 1.20 (1.08, 1.33) | 1.11 (1.01, 1.23) |
|  | 95-110 | 1223 | 86934.02 | 14.0681 | 1.04 (0.96, 1.11) | 1.01 (0.94, 1.08) |
|  | 110-125 | 1728 | 127274.94 | 13.5769 | 1 (ref.) | 1 (ref.) |
|  | 125-140 | 1899 | 138209.52 | 13.74 | 1.01 (0.95, 1.08) | 1.06 (0.99, 1.13) |
|  | 140-155 | 1297 | 89409.12 | 14.5064 | 1.07 (0.99, 1.15) | 1.11 (1.03, 1.19) |
|  | 155-170 | 808 | 48880.53 | 16.5301 | 1.22 (1.12, 1.32) | 1.23 (1.13, 1.34) |
|  | 170-185 | 518 | 27995.37 | 18.5031 | 1.36 (1.24, 1.50) | 1.35 (1.22, 1.49) |
|  | 185-200 | 324 | 16173.88 | 20.0323 | 1.48 (1.31, 1.66) | 1.45 (1.29, 1.63) |
|  | ≥ 200 | 594 | 24791.6 | 23.9597 | 1.76 (1.60, 1.93) | 1.68 (1.53, 1.84) |
| Female | ≤ 80 | 91 | 5406.99 | 16.8301 | 1.48 (1.20, 1.83) | 1.34 (1.09, 1.66) |
|  | 80-95 | 574 | 41601.01 | 13.7977 | 1.22 (1.11, 1.34) | 1.16 (1.05, 1.27) |
|  | 95-110 | 1322 | 117532.81 | 11.2479 | 0.99 (0.93, 1.07) | 0.98 (0.91, 1.05) |
|  | 110-125 | 1775 | 156943.68 | 11.3098 | 1 (ref.) | 1 (ref.) |
|  | 125-140 | 1641 | 142297.53 | 11.5322 | 1.02 (0.96, 1.09) | 1.05 (0.98, 1.12) |
|  | 140-155 | 1095 | 86088.35 | 12.7195 | 1.13 (1.04, 1.21) | 1.12 (1.01, 1.21) |
|  | 155-170 | 684 | 46345.88 | 14.7586 | 1.31 (1.20, 1.43) | 1.26 (1.15, 1.37) |
|  | 170-185 | 431 | 26664.92 | 16.1636 | 1.43 (1.29, 1.59) | 1.37 (1.23, 1.52) |
|  | 185-200 | 289 | 15960.05 | 18.1077 | 1.60 (1.41, 1.81) | 1.49 (1.31, 1.68) |
|  | ≥ 200 | 537 | 23518.56 | 22.833 | 2.02 (1.83, 2.22) | 1.84 (1.67, 2.03) |

*Hazard ratios were calculated by Cox models after adjusting for age at baseline, sex (if applicable), family income, residential area, smoking status, diabetes duration (≥5 years/< 5 year), alcohol intake, regular exercise, body mass index, systolic blood pressure, Charlson comorbidity index and total cholesterol
