# Supplementary material for: The sweet spot: fasting glucose, cardiovascular disease, and mortality in older adults with diabetes: a nationwide population-based study
Source: Cardiovasc Diabetol. 2020 Apr 1;19:44. doi: 10.1186/s12933-020-01021-8 (PMC7110776; doi:10.1186/s12933-020-01021-8)
Supplement: Supplementary file 5 — Additional file 5. Hazard ratios and 95% confidence intervals of all-cause mortality, and CVD by 10 categories of fasting glucose level at baseline. [file 12933_2020_1021_MOESM5_ESM.docx]

**Additional file 5. Hazard ratios and 95% confidence intervals of all-cause mortality, and CVD by 10 categories of fasting glucose level at baseline**

| Event | Fasting Glucose (mg/dL) | Number of event | Follow-up duration  (person-years) | Incident rate (per 1,000 person-years) | Adjusted hazard ratio*  (95% confidence interval) |
| --- | --- | --- | --- | --- | --- |
| All-cause mortality |  |  |  |  |  |
|  | ≤79 | 349 | 10637.92 | 32.81 | 1.39 (1.24-1.55) |
|  | 80-94 | 1857 | 74421.57 | 24.95 | 1.23 (1.17-1.30) |
|  | 95-109 | 4238 | 211544.25 | 20.03 | 1.08 (1.04-1.13) |
|  | 110-124 | 5260 | 293999.64 | 17.89 | 1 (reference) |
|  | 125-139 | 5373 | 290389.59 | 18.50 | 1.05 (1.01- 1.09) |
|  | 140-154 | 3671 | 182174.08 | 20.15 | 1.13 (1.08-1.18) |
|  | 155-169 | 2227 | 99423.1 | 22.40 | 1.20 (1.14-1.26) |
|  | 170-184 | 1416 | 57331.8 | 24.70 | 1.28 (1.21-1.36) |
|  | 185-199 | 992 | 33796.48 | 29.35 | 1.50 (1.40-1.61) |
|  | ≥200 | 1829 | 51389.79 | 35.59 | 1.72 (1.63- 1.81) |

| Event | Fasting Glucose (mg/dL) | Follow-up duration  (person-years) | Incident rate (per 1,000 person-years) | Adjusted hazard ratio*  (95% confidence interval) |
| --- | --- | --- | --- | --- |
| Cardiovascular events |  |  |  |  |
|  | ≤79 | 10196.6 | 17.46 | 1.17 (1.01-1.36) |
|  | 80-94 | 71565.21 | 14.85 | 1.12 (1.05-1.20) |
|  | 95-109 | 204466.84 | 12.45 | 1.00 (0.95-1.05) |
|  | 110-124 | 284218.61 | 12.33 | 1 (reference) |
|  | 125-139 | 280507.05 | 12.62 | 1.05 (1.01-1.10) |
|  | 140-154 | 175497.47 | 13.63 | 1.09 (1.04- 1.15) |
|  | 155-169 | 95226.41 | 15.67 | 1.20 (1.12- 1.27) |
|  | 170-184 | 54660.29 | 17.36 | 1.28 (1.19-1.38) |
|  | 185-199 | 32133.93 | 19.08 | 1.37 (1.25-1.49) |
|  | ≥200 | 48310.17 | 23.41 | 1.58 (1.48-1.69) |

*Hazard ratios were calculated by Cox models after adjusting for age at baseline, sex, family income, residential area, smoking status, diabetes duration (≥5 years/< 5 year), alcohol intake, regular exercise, body mass index, systolic blood pressure, charlson comorbidity index, **anti-hyperglycemic agent** and total cholesterol
